# Abrupt Reoccurrence of Acquired Postencephalitic Hemidystonia After Unexpected Discontinuation of Thalamic DBS: An Embarrassing Situation

**DOI:** 10.1002/mdc3.70586

**Published:** 2026-03-09

**Authors:** Arif Abdulbaki, Joachim Runge, Assel Saryyeva, Joachim K. Krauss

**Affiliations:** ^1^ Department of Neurosurgery Hannover Medical School Hannover Germany

**Keywords:** deep brain stimulation (DBS), hemidystonia, postencephalitic dystonia, thalamus

Hemidystonia is a phenotype of dystonia affecting only one side of the body resulting from various etiologies.^1^ Thalamic, pallidal, and subthalamic deep brain stimulation (DBS) can be considered treatment options.^2^, ^3^

While in the first 2 years of chronic DBS, dystonic symptoms might reoccur within minutes or hours after cessation of stimulation, a more delayed reoccurrence has been described after longer periods of successful DBS.^4^, ^5^ Here, we report a woman with hemidystonia who experienced abrupt reoccurrence of hemidystonia, resulting in a socially embarrassing situation.

## Case Report

**Figure 1:**
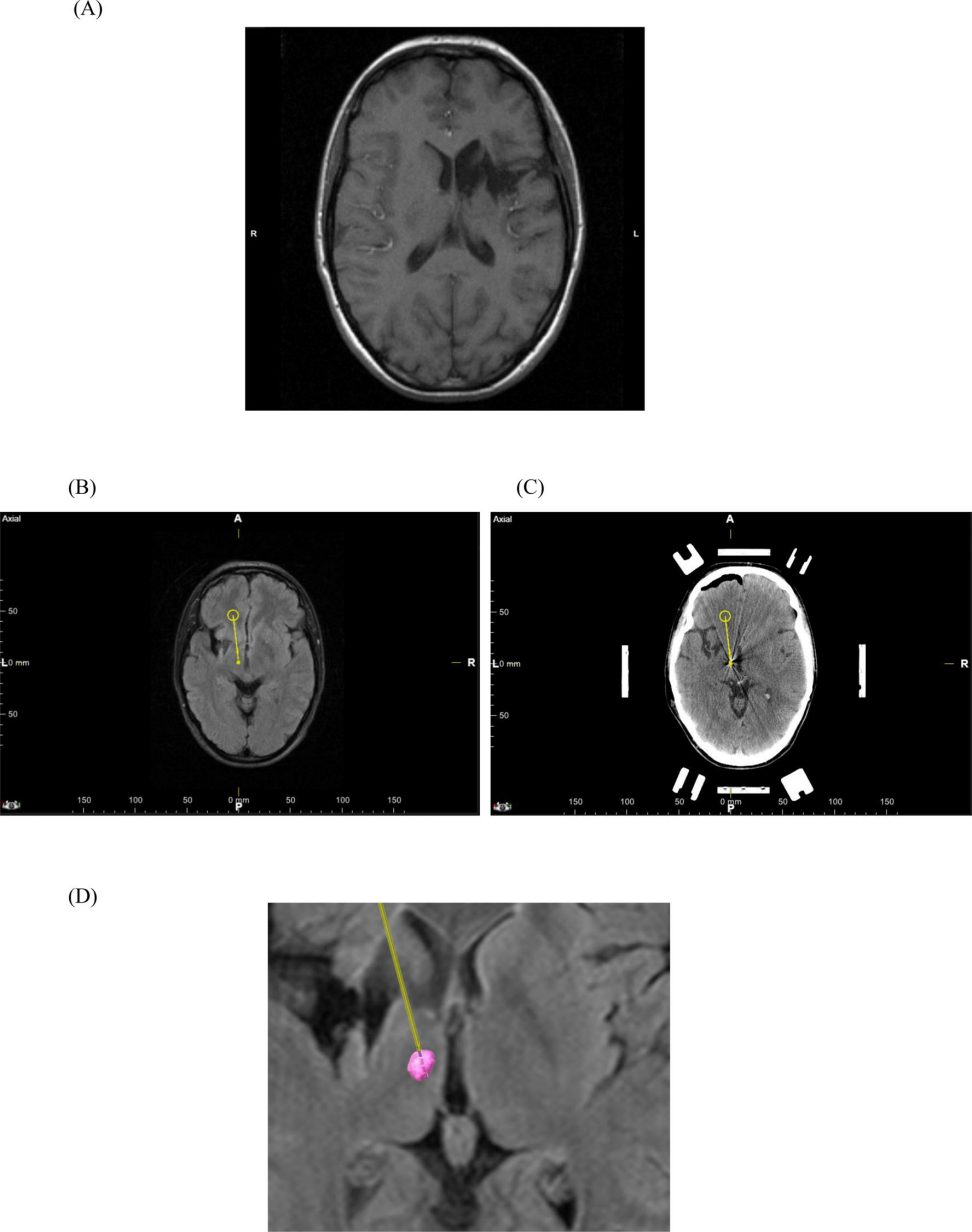
Pre‐ and postoperative imaging demonstrating structural abnormalities and electrode localization using MRI and CT. (A) Preoperative MRI (T1 sequence) shows a left‐sided lesion involving the head of the caudate, anterior putamen, and extending into the adjacent insular cortex. (B) Preoperative MRI (FLAIR sequence), with the projected position of the implanted DBS electrode targeting the ventralis intermedius thalamic nucleus (*x* = 13, *y* = −4, *z* = 0), localized through fusion with (C) the postoperative stereotactic CT scan using Brainlab software (Munich, Germany). (D) Three‐dimensional reconstruction, illustrating localization of the electrode contact within the ventralis intermedius nucleus of the thalamus.

A 35‐year‐old woman presented with right‐sided hemidystonia. At the age of five, she had herpes encephalitis, and she subsequently developed hemidystonia. MRI imaging showed a left‐sided lesion involving the caudate, anterior putamen, and extending into the adjacent insular cortex (Fig. 1A).

She underwent stereotactic implantation of a DBS electrode (Cartesia, Boston Scientific, MA, USA), targeting the ventralis intermedius thalamic nucleus connected to a rechargeable implantable pulse generator (IPG) (Vercise Gevia) (Fig. 1B,C). The Vim was chosen with regard to experiences from historic data as a target for hemidystonia.^2^, ^6^ Upon chronic stimulation, the Burke‐Fahn‐Marsden Dystonia Rating Scale (BFMDRS) motor score changed from 34 preoperatively to 18, reflecting a 47% improvement.

At a follow‐up visit, 5 years after surgery, she and her mother mentioned that she had experienced a very embarrassing situation in public. Sudden cessation of stimulation due to unexpected discharge of the IPG had resulted in reoccurrence of her hemidystonia, with elevation and extension of the right arm, resembling the “Hitler salute.” After recharging the IPG and reactivating the DBS system, dystonia improved rapidly. The rapid reoccurrence and subsequent improvement of dystonia could be reproduced when switching the DBS system off as demonstrated in Video 1.

**Video 1:** Clinical video demonstrating the patient's dystonic posture of the right arm. The first segment shows the baseline condition prior to deep brain stimulation (DBS) implantation. The second segment, recorded after 5 years of chronic DBS, illustrates alternating DBS‐On and DBS‐Off conditions. Discontinuation of chronic stimulation leads to an abrupt reoccurrence of dystonia, with elevation of the right arm, resembling a “Hitler salute.” Reactivation of stimulation results in rapid and marked improvement.

## Discussion

The dystonic posturing of the right arm had a phenomenologically different appearance before and after chronic thalamic DBS. While elevation and extension of the right arm may signify various meanings in different counties (such as showing something, or tell someone to leave), in Germany this is immediately reminiscent of the “Hitler salute.” This gesture is subject to prosecution under §86a of the German Criminal Code in Germany. Thus, the situation was not only socially embarrassing but also the patient feared possible legal consequences.

Involuntary gestures or movements with socially inappropriate or even illegal connotations have been reported in other movement disorders, such as Tourette's syndrome, where complex tics or copropraxia may signify obscene or offensive gestures.^7^ Awareness of such incidences is important for clinicians and patients to mitigate psychosocial distress and stigma.

There is limited data about the course of dystonic symptoms after discontinuation of DBS. Dystonia can also re‐occur rapidly when pallidal DBS is switched off, particularly for phasic components, which have been shown to re‐occur immediately, whereas tonic postures typically re‐occur only more gradually after a substantially longer delay of up to 120 min.^4^ In addition, after 3 years of chronic pallidal stimulation, dystonia was reported to re‐occur only after 18 months in a case of acquired dystonia.^8^ Since our observation of rapid re‐occurrence of dystonia after thalamic DBS is based on a single patient, however, no strong conclusions can be drawn. The underlying etiology of dystonia (acquired dystonia in our case) may also play a role in the variability of both the timing and characteristics of symptom recurrence after DBS discontinuation.

The long‐term effects of pallidal and thalamic DBS on neuroplasticity might differ in several aspects. A paired‐pulse DBS study demonstrated short‐term synaptic facilitation within the subthalamic–pallidal network but not with thalamic stimulation.^9^ This observation may indicate physiological differences in how these targets respond to chronic stimulation, although the mechanisms and their relevance for symptom recurrence remain uncertain. Given the presence of a structural lesion, alterations in local and network‐level neurophysiology may influence the response to stimulation and contribute to differences observed across targets. In fact, the almost immediate re‐appearance of hemidystonia in our patient with thalamic DBS is reminiscent of the phenomenon that tremor upon discontinuation of thalamic DBS reoccurs rapidly and sometimes with a rebound effect even after long‐term stimulation.^10^

Our report emphasizes the need for further research to understand the mechanisms of thalamic DBS in dystonia and the underlying phenomena contributing to the discrepancies regarding the sequelae of discontinuing chronic stimulation.

## Author Roles

(1) Research project: A. Conception, B. Organization, C. Execution; (2) Statistical Analysis: A. Design, B. Execution, C. Review and Critique; (3) Manuscript: A. Writing of the first draft, B. Review and Critique.

A.A.: 1A, 3A

J.R.: 3B

A.S.: 3B

J.K.K.: 1A, 3A

## Disclosures

**Ethical Compliance Statement:** The authors confirm that the approval of an institutional review board was not required for this work. Written informed consent was obtained from the patient for publication of this case and the accompanying video. We confirm that we have read the Journal's position on issues involved in ethical publication and affirm that this work is consistent with those guidelines.

**Funding Sources and Conflict of Interest:** AA, JR, and AS declare that there are no funding sources or conflicts of interest relevant to this work. JKK is a consultant to Boston Scientific, Medtronic, Aleva and Inomed. This case report did not receive any specific grant from funding agencies in the public, commercial or not‐for‐profit sectors.

**Financial Disclosures for the Previous 12 Months:** The authors declare that there are not additional disclosures to report.

## Financial Disclosures and Conflicts of Interest

Author disclosures are available in the Supporting Information.

## Supporting information


**Data S1.** Coi_disclosure.

## Data Availability

Data sharing not applicable to this article as no datasets were generated or analysed during the current study.
